# Preventive Palliation in the Elderly - Organizing Health Camps for the Rural Aged

**DOI:** 10.4103/0973-1075.68407

**Published:** 2010

**Authors:** Abhijit Dam, Nivedita Datta, Usha Rani Mohanty, RK Karn, Dara Singh, Sanjay Kumar

**Affiliations:** Kosish - The Hospice, Bokaro Steel City, Jharkhand, India

**Keywords:** Health camp, Rural elderly, Preventive palliation

## Abstract

Most of the needs of elders for support and assistance in the later stages of life are fulfilled by informal helpers. The position of a large number of older persons has become vulnerable due to which it cannot be taken for granted that their children will be able to look after them when they need care in old age, specially in view of the longer life span implying an extended period of dependency and higher costs to meet health and other needs. The condition of the rural elderly is even more pitiable, contrary to our beliefs, as availability, affordability and accessibility to medicare facilities are poor. We undertook the task of organizing a health camp in a rural set-up with the idea of implementing our concept of “preventive palliation” in which excellent palliative care was coupled with a pinch of prevention, like routine checks of blood pressure, routine physical check-ups, etc, so that any aberration can be detected early and necessary rectification measures can be implemented. These periods of routine check-ups can also be used to assess the psycho-social, cultural and emotional problems, if any. Such an approach, say every monthly, gives the elderly something to look forward to and ensures a high degree of customer satisfaction and greatly reduces the burden on the current health system. The challenges faced and the data obtained from this study were shocking. The elderly living in rural areas of the tribal state of Jharkhand suffer from poor physical and mental health, a factor which was rather unexpected in the Indian cultural system in the rural setting. Simple strategies like implementing routine health check ups with provision of “nutritious meal program” can go a long way in mitigating these problems in a cost-effective and simple manner. To make the government-based programs accessible and available to the end-users, participation of local bodies like NGOs is mandatory. *Preventive palliation*, a concept introduced by Kosish, is the way forward for providing palliative care to the rural-based elderly in most parts of India. This concept takes into account the local cultural, social, financial and long term feasibility and sustainability aspects of the care process.

## INTRODUCTION

It is a fact that most of the needs of elders for support and assistance in the later stages of life are fulfilled by informal helpers like family, neighbors and friends. It is true that family ties in India are very strong and an over whelming majority live with their sons or are supported by them. Also, working examples find the presence of old persons emotionally bonding and of great help in managing the household and caring for children. However, due to the operation of several factors, the position of a large number of older persons has become vulnerable due to which they cannot be taken for granted that their children will be able to look after them when they need care in old age, specially in view of the longer life span implying an extended period of dependency and higher costs to meet health and other needs.

## CHRONIC ILLNESS AMONG THE ELDERLY

Most elderly people experience some chronic conditions. For elderly people, living with chronic conditions can resemble walking at the edge of a cliff. The slightest blow - such as a cold or the flu - will stress their already fragile systems and might push them over the edge. Very often, the health care system will label this final blow as the cause of death, when the *cause* was more accurately the cumulative effect of illnesses or frailty. However, predicting the timing of the “big fall” is often difficult.[1] Those with serious chronic illness may live reasonably well for many years or succumb quickly to early complications.

Lynn and Adamson in the Rand Health White Paper, have split these conditions into three categories, viz.,

### Nonfatal chronic Illness

Common nonfatal chronic conditions include arthritis and hearing or vision problems. Most elderly people live for many years with these conditions, which gradually worsen but seldom pose a threat to life.

### Serious, eventually fatal, chronic conditions

An important subset of chronic conditions, however, tends to worsen and eventually cause death. The common fatal chronic conditions are cancers, organ system failures, dementia, and strokes.

### Frailty

*Frailty* is the fragility of multiple body systems as their customary reserves diminish with age and disease. Frailty may already be a major path through the end of life, but the standard classifications of illness often fail to recognize it. Likewise, much of how doctors and nurses think is organized around diagnosis, and this drives the course of care and treatment. However, chronically ill people coming to the end of life ordinarily have multiple diagnoses, none of which may be particularly revealing about aggregate severity of illness.

## FLAWS IN OUR EXISTING MEDICAL SYSTEM

The medical fraternity is organized around diagnosis and cure, which is often not possible, especially in the elderly nearing the end of life and having multiple diagnoses. Furthermore, a specific diagnosis may not shed light on their needs. For example, a person may have greater need for help in daily functioning - grocery shopping or in-home supervision - than for a particular course of medical treatment. People living with serious illness at the end of life can be identified not from certainty of timing of death, but from “living on thin ice” - suffering long periods of illness or disability, diminished functioning, and potential exacerbation of symptoms, any of which could prove fatal. They could keep “living on thin ice” for some years, or die in a week.[1]

Hence the need of the hour in such patients is excellent palliative care often coupled with a pinch of prevention, like routine checks of blood pressure, blood sugar levels, routine physical check-ups, etc, so that any aberration can be easily detected and necessary rectification measures can be implemented. These periods of routine check-ups can also be used to assess the psycho-social, cultural and emotional problems, if any. Such an approach, say every monthly, gives the elderly something to look forward to and ensures a high degree of customer satisfaction and greatly reduces the burden on the current health system.

## PREVENTIVE PALLIATION IN THE ELDERLY

### The rural elderly

The rural based elderly are indeed a special population. Rural elders are cared for by their families-and to a degree that exceeds their urban counterparts. The first part of this assumption is certainly true, rural elders do receive a great deal of assistance from their families, neighbours and friends; but there is no credible evidence that they are particularly *advantaged* in this regard. Rural elders may however have to rely on informal networks more heavily because of their location in areas where the formal service network is less available.[2]

Organizing a health camp exclusively for the elderly in a rural set-up poses a challenge. The first challenge is to get the message to the people about the date and location of the proposed health camp. In Jharkhand, villages are isolated with people of different castes like *bhumiyars, telis, bauris*, etc. and different tribes like *santhals, mundas, oraons, khariyas*, etc. It is often so that people of different villages do not see eye-to-eye with each other. Getting them to approach a common platform for healthcare provision is understandably difficult. The main hurdle is to get them to come out of their houses and travel a distance to attend the camp. The elders in the rural areas too are a neglected lot contrary to our understanding in the Indian culture. The bread winners of the family have no time to spare to get them to the camp and the onus of responsibility is often left to the lady of the house (who is busy with routine household chores). It is often the grandchildren who support the elderly and help them come to the camp. But then, if you do not have access to funding and hence cannot arrange for transport for the elderly, as in our case, then you have to offer some juicy incentive for them to take all the trouble to attend the camp. Free medicines is an incentive for the elderly but what about the little children who act as guardians of the elderly accompanying them and physically supporting them?

**Figure 1:**
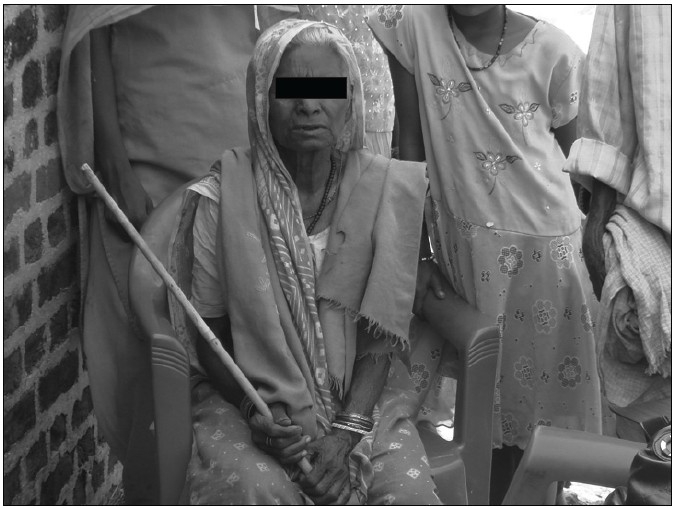
An elderly lady attending the camp with her little granddaughter, who is acting as her guardian, the elderly are in their second chlidhood

Hence we arranged the “*once a month nutritious meal*” program, which would act as an incentive to get the elderly as well as the children accompanying them to attend the camp. And lo and behold, on the day of the camp we saw the elderly trudging down supported by their grandchildren [Figure 1]. And then to imbibe the importance of community participation in them, we invited the children to participate in the process of preparing the food, which they readily did with gusto. However, we were saddened to see that the upper caste elderly, especially the women, refused to sit and eat with the villagers of lower caste. It is an unfortunate reality that the caste system is still prevalent in India, especially in the rural areas.

**Figure 2:**
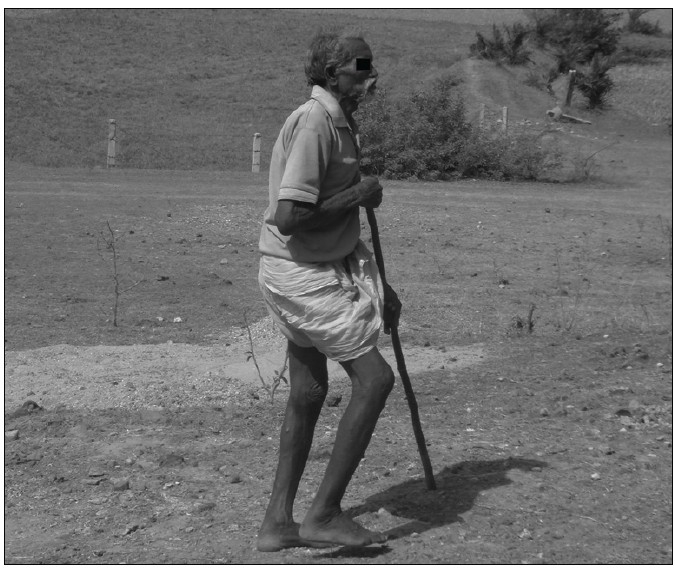
Giving back a dignified life to them is our aim

The program was a hit with enquiries pouring in as to when the next camp would be organized [Figure 2].

The camp was run by two doctors, 3 nurses and 2 paramedics during which more than 150 patients were seen. Considering that the ambient temperature being 39°C, this was a significant number. In addition the SF 8 Health Questionnaire was used to evaluate the health status of the elderly.

The most common ailments that we came across were aches and pains, undiagnosed hypertension and visual problems. Undiagnosed hypertension and diabetes mellitus pose a significant problem and timely detection and initiation of treatment can prevent mortality and significant morbidity like cerebrovascular accidents and coronary events. These also impose a significant burden on the healthcare resources as well as financial and social burden on the family.

It was saddening to observe that in spite of the excellent policy framework by the Government for the elderly, hardly any benefit reaches the clients. Implementation of the policy is an altogether different matter. We were surprised by the fact that our NGO, Kosish, in spite of having no grants whatsoever, could achieve a lot and wondered where the provisions for vehicles, medicines, old age homes, etc by the government and other charitable bodies actually landed up! In the nearly 10 years since the formation of the Jharkhand state, ours was the first health camp for the elderly to be conducted at the rural level in the district of Bokaro!

We encountered a larger number of elderly females than males, thereby confirming the fact that in the age group of more than 60 years, females had a greater life span [Table 1]. This paradoxically poses a burden on the society, especially in rural India as most of the ladies above 60 years of age are illiterate and totally dependent financially and socially on their husbands or sons. In the Indian cultural system, the married daughters usually do not take care of their parents;the onus of responsibility is left to the sons and grandchildren. The condition of the elderly widows is even more pitiable with neglect and abuse being common.

**Table 1:** Demographic data of rural elderly at Pindrajhora

N = 150, average age: 63.15 years	Males	Females
Number	54	96
Weight (kg)	60.7	45.6
Height (m)	1.61	1.49
Body mass index	23.4	20.5
Undiagnosed hypertension (using a cutoff of 140/90 mm Hg)	18	30
Visual problems	12	28
Bodily pain affecting quality of life	32	70
Emotional problems(feeling anxious, depressed, irritable, low energy) in past 4 weeks	30	63
Pallor	16	50
Clinical evidence of vitamin deficiency	9	20
Chronic cough and respiratory problems	38	11

## THE SURVEY

To have an idea of the health status of the elderly, we used the SF-8 health survey. The SF health surveys are the most widely used tools in the world for measuring patient-reported outcomes, with more than 76,000,000 surveys taken. It has only eight questionnaire items.[3] The SF-8™ provides the long awaited “missing link” - a practical tool for directly linking the norms from large population surveys with the results from more focused clinical trials, outcomes research studies, and monitoring efforts in everyday clinical practice. Also this survey is a useful tool to measure the health related quality of life (HRQF) in a population.[4]

When the average sores in each category were applied to the SF-8 health survey for the rural elderly, we were astonished by the results! The results stated that-

1. Compared to the general population (USA)
  - Physically, your….
    - Functioning is worse
    - Pain is the same
    - Performance of work, home or other activities is worse
  - Emotionally,
    - Bothered more than most
    - Participation in social activities is about the same
    - Performance of work, home and other activities is limited much more
  - Overall, your,
    - Rating of health is worse
    - Energy level is lower
2. The physical health summary was 40, with the US average being 50.
3. The mental health summary was 39, with the US average being 50.

## CONCLUSION

These shocking data reveal that the elderly living in rural areas of the tribal state of Jharkhand not only suffer from poor physical health (a factor which is amplified by poverty and resultant malnutrition) but also from poor mental health, a factor which was rather unexpected in the Indian cultural system in the rural setting. Simple strategies like implementing routine health check ups with provision of “nutritious meal program” can go a long way in mitigating these problems in a cost-effective and simple manner. To make the government based programs accessible and available to the end-users, participation of local bodies like NGOs is mandatory.

## PREVENTIVE PALLIATION

A concept introduced by Kosish, is the way forward for providing palliative care to the rural based elderly in most parts of India. This concept takes into account the local cultural, social, financial and long term feasibility and sustainability aspects of the care process.
